# Vitamin D Supplementation in Multiple Sclerosis: A Critical Analysis of Potentials and Threats

**DOI:** 10.3390/nu12030783

**Published:** 2020-03-16

**Authors:** Julia Feige, Tobias Moser, Lara Bieler, Kerstin Schwenker, Larissa Hauer, Johann Sellner

**Affiliations:** 1 Department of Neurology, Christian Doppler Medical Center, Paracelsus Medical University, 5020 Salzburg, Austria; j.feige@salk.at (J.F.); t.moser@salk.at (T.M.); l.bieler@salk.at (L.B.); k.schwenker@salk.at (K.S.); 2 Department of Psychiatry, Psychotherapy and Psychosomatic Medicine, Christian Doppler Medical Center, Paracelsus Medical University, Salzburg; l.sellner@salk.at; 3 Department of Neurology, Klinikum rechts der Isar, Technische Universität München, 81675 München, Germany; 4 Department of Neurology, Landesklinikum Mistelbach-Gänserndorf, 2130 Mistelbach, Austria

**Keywords:** multiple sclerosis, vitamin D, intoxication, renal failure, clinical trials

## Abstract

Multiple sclerosis (MS) is a chronic inflammatory demyelinating and neurodegenerative disease of the central nervous system (CNS). In recent years, vitamin D has gained attention, as low serum levels are suspected to increase the risk for MS. Cholecalciferol supplementation has been tested in several clinical trials, since hypovitaminosis D was linked to higher disease activity and may even play a role in long-term outcome. Here, we review the current understanding of the molecular effects of vitamin D beyond calcium homeostasis, the potential beneficial action in MS and hazards including complications of chronic and high-dose therapy. In clinical trials, doses of up to 40,000 IU/day were tested and appeared safe as add-on therapy for short-term periods. A recent meta-analysis of a randomized, double-blind, placebo-controlled clinical trial investigating vitamin D as add-on therapy in MS, however, suggested that vitamin D had no therapeutic effect on disability or relapse rate. We recognize a knowledge gap for chronic and high-dose therapy, which can lead to life-threatening complications related to vitamin D toxicity including renal failure, cardiac arrythmia and status epilepticus. Moreover, vitamin D toxicity may manifest as fatigue, muscle weakness or urinary dysfunction, which may mimic the natural course of progressive MS. Given these limitations, vitamin D supplementation in MS is a sensitive task which needs to be supervised by physicians. While there is strong evidence for vitamin D deficiency and the development of MS, the risk-benefit profile of dosage and duration of add-on supplementation needs to be further clarified.

## 1. Introduction

Multiple sclerosis (MS) is a chronic inflammatory and degenerative disease of the central nervous system (CNS), which usually starts in early adulthood [1]. The disease may start either with a relapsing or progressive course, and continues to be the leading cause of non-traumatic disability in young adults in Western countries. Moreover, life expectancy in the MS population is reduced by 7 to 14 years compared with the general, healthy population [2,3].

In early stages of the disease, inflammation is more evident than neurodegeneration. Over time, when entering the progressive phase of the disease, these mechanisms go into reverse [4]. Inflammation in MS is considered to be mostly driven by T-cells; however, with the introduction of B-cell depleting therapeutic agents, there is increasing evidence for CD20-expressing T- and B-cells as the second pillar in the pathogenesis of MS [5,6]. Immunotherapies have been widely used in MS, as they have been shown to reduce the relapse rate and the accumulation of new brain lesions in patients with relapsing–remitting MS (RRMS) [7,8]. In recent years, vitamin D was brought into focus as it is suspected to play an important part in the pathogenesis of MS. Several studies have been conducted to ascertain its presumed protective effects and its ability to decrease the risk of MS, disease activity and progression. Therefore, increasing attention is being paid to its potential use as an add-on therapy.

In this review, we provide an update on the current understanding of the molecular effects of vitamin D beyond regulating calcium homeostasis, and summarize the current study evidence for vitamin D supplementation in MS. Moreover, we discuss potential hazards, including the complications of chronic and high-dose therapy. Of note, Vitamin D is a fat-soluble vitamin which needs to undergo two conversion steps to become active. First, it is converted to calcidiol (25(OH) D) in the liver converted to calcitriol (1,25(OH) D_2_), mostly in the kidneys. Calcitriol is the active, steroid-hormone form of vitamin D and interacts with the vitamin D receptor (VDR). As a medication, cholecalciferol may be taken as a dietary supplement to prevent or to treat vitamin D deficiency. Calcidiol is measured in serum as it best reflects vitamin D status.

## 2. Multiple Sclerosis and Vitamin D

### 2.1. Vitamin D and the Pathogenesis of Multiple Sclerosis

The etiology of MS remains elusive. There is increasing evidence that an interplay of genes and environmental factors determines the risk of developing the disease [9,10]. Regarding the potential environmental factors, a number of observational studies disclosed the risk of developing MS in the setting of low serum levels of 25(OH)D. The largest study to date evaluated prospective data from the US military repository of over 7 million military personnel and found an inverse correlation between vitamin D serum levels and risk of developing MS. High circulating levels of vitamin D were therefore propagated as a potential protective factor [11]. Vitamin D seems to be not only an environmental risk factor contributing to the risk of developing MS but also able to modulate disease activity and progression. In several studies, low vitamin D serum levels have been associated with an increased relapse rate, increasing disability and an increased lesion load, as seen on magnetic resonance imaging (MRI) [12,13,14].

### 2.2. Effects of Vitamin D on the Immune System

Vitamin D is best known for the regulation of calcium physiology, as it stimulates intestinal calcium absorption [15]. In addition, vitamin D is critical for brain development and function, cell proliferation and apoptosis, regulation of blood pressure, insulin secretion and the differentiation of immune cells and modulation of immune responses [16,17,18,19]. Vitamin D and its metabolites clearly alter the phenotype and function of various immune cells in vitro. The vast majority of these immune modulatory properties are exerted via interaction with the VDR [20].

There emerging evidence that vitamin D plays an important role in the pathogenesis of MS due to its role in lymphocyte activation and proliferation, T-helper cell differentiation and its regulatory effects on immune response [21]. Moreover, the innate system response is promoted and the adaptive immune activity is subdued by suppressing the production of inflammatory cytokines mediated by type 1 T-helper cells (Th1) by vitamin D [22]. During vitamin D supplementation, levels of interleukin-10 (IL-10) and IL-17 changed significantly in several studies [23,24]. High doses of vitamin D were also able to reduce the proportion of IL-17-producing CD4^+^ T-cells and increase central memory CD4^+^ T-cells and naïve CD4^+^ T-cells [25]. Monocytes cultured in the presence of 1,25(OH)_2_D_3_ showed a VDR-dependent loss of MHCII and a reduction in co-stimulatory molecules, such as CD40, CD80 and CD86 [20]. In addition, monocytes showed a diminished release of Th1 and Th17 cell-inducing cytokines, such as IL-12 and IL-23, whereas the production of IL-10 and CCL22, involved in regulatory T cell and Th2 immune responses, was enhanced. The exposure of B cells to 1,25(OH)_2_D_3_ inhibited their proliferation, plasma cell differentiation including immunoglobulin G and -M secretion, memory B cell generation and induced B cell apoptosis in proliferating B cells [26,27]. These findings, however, are challenged by a recent report which suggests that vitamin D at moderate levels may exert a direct immunoregulatory effect, while continuous high-dose vitamin D treatment might exacerbate clinical disease activity by raising levels of T-cell-excitatory calcium [28].

Furthermore, a possible interaction between Epstein–Barr virus (EBV) and vitamin D serum levels has been proposed. Infection with EBV during or shortly after childhood has long been suspected as a key environmental risk factor in MS for many years [29]. Higher levels of anti-EBV nuclear antigen-1 (EBNA-1) immunoglobulin (Ig) G are associated with an increased risk of developing MS, and quicker accumulation of neurological disability and a higher rate of disease activity. In this regard, prior to MS onset, anti-EBNA-1 IgG levels increased, whereas vitamin D serum levels are decreased. During vitamin D supplementation in patients with clinically definite MS, anti-EBNA-1 IgG levels are significantly reduced [30]. This may implicate clinical relevance, since higher anti-EBNA-1 IgG levels might be associated with an increase in the number of active MRI lesions.

Some modulatory effects of vitamin D on B-cells have also been suggested, such as the inhibition of plasma cell generation, inhibition of T-cell co-stimulation and enhancement of regulatory B-cell activity [31]. In patients with hypovitaminosis D, MS-related alterations in B-cells are more pronounced compared to patients with normal vitamin D serum levels and recede after vitamin D supplementation [32].

### 2.3. Randomized-Controlled Trials of Vitamin D as Add-On Therapy in Multiple Sclerosis

In recent years, several studies have been conducted to investigate the possible beneficial effects of vitamin D supplementation in patients with MS. Results were contradictory: some studies confirmed a reduction in disease activity and severity [33,34,35,36], while others did not report favorable outcomes [37,38,39,40].

In the clinical trials which are summarized in Table 1, doses ranging from 10,000 to 40,000 international units (IU)/day were tested and appeared safe as an add-on therapy. In 2018, a Chinese study group conducted a meta-analysis to evaluate the efficacy of vitamin D supplementation in MS patients [41]. No significant beneficial effect on Expanded Disability Status Scale (EDSS) and annualized relapse rate (ARR) was found. However, only six studies were analyzed, of which sample sizes were mostly small, and study designs varied, which impedes direct comparison. Another meta-analysis which reviewed 13 studies of higher quality, reported a modest decrease in both relapse rate and radiological activity during vitamin D supplementation, whereas the effect on disability progression remained unclear [42]. In regard to this, a different meta-analysis of six studies was conducted in 2019 in which the effect on EDSS progression was reviewed, which did not find any effect of vitamin D supplementation on disability progression [43].

The results of the three most recent randomized controlled trials (RCTs) were published in 2019 and 2020, respectively. The SOLAR trial studied the effect of high-dose vitamin D supplementation (14,007 IU/d) vs. a placebo as an add-on therapy to interferon beta-1a in 229 patients, of which 113 received the study drug [44]. No increase in median 25(OH)D levels was found in the placebo group at week 48, whereas an approximately 4-fold increase in 25(OH)D levels was observed in the high-dose vitamin D3 group. The primary endpoint of having “no evidence of disease activity” (NEDA)-3, which is defined as no relapses, EDSS progression or combined unique active lesions, after 48 weeks was not reached even though the vitamin D group had better MRI outcomes. Treatment-emergent AEs (TEAEs) were reported by 87.6% and 80.2% of patients in the high-dose vitamin D3 and placebo groups, respectively. The majority of TEAEs were unrelated to treatment, and those possibly or probably related to treatment were reported at comparable frequency between the groups. Neurofilament light (NfL) chains were analyzed in a substudy, but supplementation of high-dose vitamin D3 for 48 weeks was not associated with lower NfL levels [45].

The CHOLINE trial (“Cholecalciferol in relapsing-remitting MS: A randomized clinical trial”) evaluated the effect of high-dose vitamin D (100,000 IU every other week) vs. a placebo as add-on treatment to interferon beta-1a in 181 patients for 96 weeks [46]. The primary endpoint of a significant reduction in terms of ARR was not met, whereas a slight trend of ARR reduction, better MRI outcomes and lower progression of EDSS was reported in the vitamin D group. There was a rate of 16.7% for TEAE but no statistical difference between the two groups.

The EVIDIMS trial (“Efficacy of vitamin D supplementation in multiple sclerosis”) compared the effects of a high- (20,400 IU) versus low-dose (400 IU) of cholecalciferol supplementation every other day on the clinical and imaging markers of disease activity as an add-on to interferon-β1b [47]. Mean 25OH vitamin D serum level (ng/mL) were 65.0 (standard error of the mean, SEM 5.5) for the high dose and 22.3 (SEM 1.4) for the low dose. The changes from baseline were 45.9 (SEM 5.4) and 5.9 (SEM 2.3), respectively. The majority of adverse events were mild or moderate and not considered to be related to the study medication. No serious adverse events and no vitamin-D-related toxicity was recorded. After 18 months, clinical (relapse rates, disability progression) and radiographical (T2-weighted lesion development, contrast-enhancing lesion development, brain atrophy) did not differ between both treatment arms. Post-study power calculations suggested that the sample size (*n* = 53 for randomized patients) was too low to prove the hypothesis.

Of note, the US Institute of Medicine (IOM) issued that doses equal to or above 50,000 IU/day for several weeks or months are frequently associated with toxic side effects [54]. Therefore, time seems to be a limiting factor in the two dose-escalation trials evaluating ultra-high doses vitamin D in MS, which did not find evidence for vitamin D toxicity [37,38].

### 2.4. High-Dose Vitamin D Supplementation in Multiple Sclerosis

#### 2.4.1. Lack of Clinical Trials for Ultra-High Dose Supplementation

In clinical practice, an increasing number of patients request the evaluation of their vitamin D status and want to know whether they should use any kind of supplementation. We increasingly notice patients which ingest ultra-high doses of vitamin D of up to 80,000–100,000 IU daily due to information found on the internet. This trend can be traced back to the so-called “Coimbra protocol” (http://t1p.de/sw3u), which promises a cure for MS by the intake of extremely high dosages ranging from 50,000-300,000 IU together with dietary recommendations and guidance by a Coimbra-certified medical doctor. However, this protocol is not based on study evidence and can cause life-threatening complications.

#### 2.4.2. Vitamin D Toxicity: A Life-Threatening Complication

Vitamin D toxicity resulting from excessive intake of vitamin D is characterized by hypercalciuria, hypercalcemia, elevated 25(OH)D > 150 ng/mL (>375 nmol/L), and usually normal or slightly increased 1,25(OH)_2_D concentration. There is a broad spectrum of clinical manifestations of vitamin D toxicity, but they are mostly related to hypercalcemia. Neuropsychiatric manifestations include cognitive disturbances, confusion, apathy, drowsiness, depression, psychosis, and, in extreme cases, a stupor and coma [55]. The gastrointestinal symptoms can range from recurrent vomiting, abdominal pain, polydipsia, anorexia, constipation and peptic ulcers to pancreatitis. Among the cardiovascular manifestations hypertension, shortened QT interval, ST segment elevation, and bradyarrhythmia with first-degree heart block on the electrocardiogram have been observed. The renal symptoms include hypercalciuria as the earliest sign, and polyuria, polydipsia, dehydration, nephrocalcinosis, and renal failure as the end stage [56]. Other reported symptoms of vitamin D toxicity caused by hypercalcemia are band keratopathy, hearing loss, and painful periarticular calcinosis.

Galior summarized cases of vitamin D toxicity related to the overcorrection of vitamin D deficiency and emphasizes that vitamin D levels above 150 ng/mL are associated with toxicity and must be avoided [57]. The doses ranged from 50,000 to 2.604,000 IU/day, which led to vitamin D levels between 150–1220 ng/mL and marked hypercalcemia with serum calcium concentrations between 11.1–23.1 mg/dl. The side effects reported were partly life-threatening side effects and included renal failure, cardiac arrhythmias, calcification of coronary vessels and heart valves and death. Milder manifestations of vitamin D toxicity included muscle weakness, hypertension, neuropsychiatric disturbances, gastrointestinal upset, polyuria and polydipsia. There is also a case unrelated to MS where a 67-year-old woman had taken 600,000 IU (rather than the intended 600 IU) of cholecalciferol daily for more than 3 years because of a compounding error by the pharmacy. She had reversible hypercalcemia and only partially reversible kidney injury [58].

There is limited information available concerning vitamin D toxicity in MS patients. A collection of 21 patients from Brazil who were supplemented with an average dose of 87,000 IU cholecalciferol daily over an average period of one year is the largest cohort to date. Notably, 17 of 21 patients had clinical disease activity in terms of relapses, new MRI lesions or an increase in disability. Moreover, direct side effects of vitamin D supplementation, such as gastric symptoms, seizures, severe hypercalcemia, kidney failure, nephrolithiasis and nephrocalcinosis were noted [59]. The side effects seen in our patient with uncontrolled intake of ultra-high doses of vitamin D which mimicked the progression of primary progressive MS is discussed in the following chapter [60].

#### 2.4.3. Vitamin D Toxicity May Mimic Progressive Multiple Sclerosis

Progressive MS is a clinical form of the disease characterized by gradual accrual of disability independent of relapses over time [61]. Recently, the CD20-depleting antibody ocrelizumab and the S1P1 receptor antagonist siponimod have been approved for the treatment of this patient subgroup. Yet, there are significant unmet needs in the treatment of progressive MS, which relate in part to an incomplete understanding of the disease pathogenesis and a lack of validated outcome measures. Gradual change in functional ability over time most often relates to walking. Signs and symptoms include a worsening of sensory and motor symptoms, progressive paraparesis, fatigue, muscle weakness, stiffness, pain, bladder dysfunction, constipation and cognitive impairment [62]. Of note, there is increasing evidence for the occurrence for cardiac autonomic dysfunction in MS [63]. Thus, a considerable overlap of symptoms of vitamin D toxicity is present, as shown in Table 2.

Indeed, we reported a patient with primary progressive MS who presented with generalized weakness and fatigue, which was caused by hypercalcemia after the uncontrolled intake of more than 50,000 IU of cholecalciferol per day over several months [60]. Several treatment strategies and intensive care unit (ICU) admission were required to achieve normocalcemia, yet renal failure persisted. Thus, the dynamics of the clinical course of progressive might be similar to that of acute or chronic vitamin D toxicity, and therefore lead to delayed diagnosis until the side effects of vitamin D supplementation are irreversible or even fatal.

## 3. Conclusions

There is emerging evidence that vitamin D plays an important role in the pathogenesis and course of neuroinflammatory diseases, including MS. Therefore, several studies have investigated the potential beneficial effects by means of vitamin D supplementation. Of note, the study duration of the clinical trials evaluating the effect of vitamin D supplementation was relatively short, with small sample sizes and, in many cases, not even placebo-controlled. The results of these trials, however, are divergent, and conclusions as to whether regular vitamin D intake is reasonable beyond the correction of hypovitaminosis D cannot be made at the moment. From a physiological viewpoint, vitamin D serum levels should reach approximately 130 ng/mL in order to exert probable therapeutic effects. We present data from clinical trials, where doses of vitamin D ranged from 10,000 to 40,000 IU/day and which appeared safe as an add-on therapy. The side effects during trials were usually minor and manageable. Of note, there is increasing evidence for potentially life-threatening complications caused by regular an ultra-high dose intake of vitamin D. Such therapeutic approaches should not take place outside of trials and may even be harmful by exacerbating autoimmune processes. The current studies were underpowered and larger randomized-controlled trials are required to determine the effect of high-dose vitamin D supplementation in MS. Such studies with stratification for the evaluation of a potential benefit at different disease stages are eagerly awaited and may require more sensitive MRI than the clinical endpoint. Of note, severe side effects might be detected only after a latency period of several weeks or even months, and require further follow-up even after termination of the study.

Taken together, there is no doubt about the clinical need to identify and correct vitamin D insufficiency with supplementation at recommended doses. The IOM states that chronic supplementation in adults should not exceed 600 IU/day (15 μg/day). Whether patients with MS and normal vitamin D levels, however, benefit from supplementation remains to be answered. We also emphasize that patients and physicians need to be aware of the potential side effects of vitamin D supplementation, including signs and symptoms mimicking clinical progression. Moreover, there certainly is no evidence of using vitamin D as a monotherapy to prevent MS or interrupt ongoing immunotherapies.

## Figures and Tables

**Table 1:** Characteristics of studies assessing efficacy and safety of vitamin D supplementation. IU: international units. EDSS: expanded disability status scale. ARR: annualized relapse rate.

Authors, Year	*n* =	Study Period	Intervention		Sex (Male/Female)		EDSS at Baseline		ARR at Baseline		Side Effects in Vitamin D Treated Patients	
			*Treatment Group*	*Control*	*Treatment Group*	*Control*	*Treatment Group*	*Control*	*Treatment Group*	*Control*	*Mild to Moderate*	*Severe*
Laursen et al. (2016) [33]	134	1 year	2000 IU/d vs. 3000 IU/d vs. 4000 IU/d	no control group	39/95	-	3.5	-	0.6	-	n.a.	n.a.
Soilu-Hänninen et al. (2012) [34]	66	1 year	20,000 IU/d	placebo	13/21	12/20	2.0	1.5	0.49	0.52	29.4%	2.9%
Etemadifar et al. (2015) [36]	15	24–28 weeks	50,000 IU/w	no treatment	0/6	0/9	1.2	1.3	1.3	1.1	none	none
Wingerchuk et al. (2005) [48]	15	48 weeks	28,000 to 280,000 IU/week	no control group	3/12	-	1.9	-	n.a.	-	53.3%	none
Burton et al. (2010) [38]	49	52 weeks	40,000 IU/d (for 28 weeks) + 10,000 IU/d (for 12 weeks) + 0 IU	<4000 IU/d	4/21	5/19	1.5	-	0.44	-	none	none
Sotirchos et al. (2015) [49]	40	6 months	10,400 IU/d	800 IU/d	5/14	7/14	3.0	2.5	n.a.	n.a.	15%	none
Stein et al. (2011) [39]	23	6 months	6000 IU/d	1000 IU/d	4/7	3/9	2.5	2.0	1.0	1.0	4.4%	none
Bhargava et al. (2014) [50]	172	96 weeks	5000 IU/d	600 IU/d	n.a.	n.a.	n.a.	n.a.	n.a.	n.a.	n.a.	n.a.
O’Connell et al. (2017) [51]	29	24 weeks	5000 vs. 10,000 IU/d	placebo	9/13	1/6	0.9	0.4	n.a.	n.a.	none	none
Golan et al. (2013) [24]	45	1 year	4370 IU/d	800 IU/d	5/19	8/13	2.9	3.6	0.28	0.38	none	none
Kampmann et al. (2012) [52]	68	96 weeks	20,000 IU/week + 500 mg calcium/d	500 mg calcium/d	11/24	9/24	2.61	2.27	0.11	0.15	n.a.	n.a.
Mosayebi et al. (2011) [53]	58	6 months	300,000 IU/month	placebo	9/17	8/25	n.a.	n.a.	n.a.	n.a.	n.a.	n.a.
Shaygannejad et al. (2012) [40]	50	1 year	0.5 µg/d	placebo	3/22	3/22	1.6	1.7	1.04	1.04	72%	none
Hupperts et al. (2019)–SOLAR [44]	229	48 weeks	14,007 IU/d	placebo	37/76	37/79	n.a.	n.a.	0.91	0.86	21.2%	none
Camu et al. (2019)–CHOLINE [46]	181	2 years	100,000 IU every other week	placebo	13/50	27/39	1.66	1.22	0.97	0.91	9.8%	3.1%
Dörr J et al. (2020)–EVIDIMS [47]	41	18 months	20,400 IU every other day	400 IU every other day	8/20	8/17	2.0	2.5	n.a.	n.a.	77.4%	none

**Table 2:** Overlap of symptoms of progressive multiple sclerosis and acute or chronic vitamin D intoxication. Symbols: - absent, (+) rare, +–+++ common to very common. MS: multiple sclerosis.

Symptoms	Progressive MS	Hypervitaminosis D
Gastric symptoms (diarrhea, constipation)	+	+++
Bladder dysfunction (polyuria, incontinence)	+++	+
Renal dysfunction	-	++
Muscle weakness	+++	++
Chronic pain (various locations)	+	+
Fatigue	+++	++
Neuropsychiatric disturbances/cognitive impairment	++	+
Altered sensorium	+++	+
Progressive paraparesis	+++	-
Gait disturbances	+++	+
Seizures	(+)	(+)
Cardiac symptoms	+	++
